# “Early Rupture of Membranes” during Induced Labor as a Risk Factor for Cesarean Delivery in Term Nulliparas

**DOI:** 10.1371/journal.pone.0039883

**Published:** 2012-06-29

**Authors:** Seung Mi Lee, Jeong Woo Park, Chan-Wook Park, Bo Hyun Yoon

**Affiliations:** 1 Department of Obstetrics and Gynecology, Seoul National University College of Medicine, Seoul, Republic of Korea; 2 Department of Obstetrics and Gynecology, Seoul Metropolitan Government Seoul National University Boramae Medical Center, Seoul, Republic of Korea; The Ohio State Unversity, United States of America

## Abstract

**Objective:**

To determine if “early rupture of membranes” (early ROM) during induction of labor is associated with an increased risk of cesarean section in term nulliparas.

**Study Design:**

The rate of cesarean section and the timing of ROM during the course of labor were examined in term singleton nulliparas whose labor was induced. Cases were divided into 2 groups according the timing of ROM: 1)“early ROM”, defined as ROM at a cervical dilatation<4 cm during labor; and 2) “late ROM”, ROM at a cervical dilatation≥4 cm during labor. Nonparametric techniques were used for statistical analysis.

**Results:**

1) In a total of 500 cases of study population, “early ROM” occurred in 43% and the overall cesarean section rate was 15.8%; 2) patients with “early ROM” had a higher rate of cesarean section and cesarean section due to failure to progress than did those with “late ROM” (overall cesarean section rate: 24%[51/215] vs. 10%[28/285], p<0.01; cesarean section rate due to failure to progress: 18%[38/215] vs. 8%[22/285], p<0.01 for each) and this difference remained significant after adjusting for confounding variables.

**Conclusion:**

“Early ROM” during the course of induced labor is a risk factor for cesarean section in term singleton nulliparas.

## Introduction

Induction of labor is one of the most common practices in obstetrics. More than 20% of pregnant women are delivered after the induction of labor, and the overall rate of induction of labor in the United States has become more than doubled from 1990 to 2006 [1]. The induction of labor is usually performed as a therapeutic option when the benefits of expeditious delivery outweigh the risk of continuing pregnancy. However the patient should be counseled about the increased risk of cesarean delivery, especially in nulliparous women[2]–[10].

For this reason, previous investigators have tried to identify risk factors for cesarean deliveries during the induction of labor. Nulliparity, advanced gestational age, increased birthweight, and use of cervical ripening agent have been reported as risk factors for cesarean delivery [8], [11]. And, on the basis of these risk factors, several investigators offered scoring system for risk of cesarean section during the induction of labor 12–16.

However, there is little information on the timing of ROM during induced labor at term as a risk factor for cesarean delivery. We have recently demonstrated that spontaneous early rupture of membranes (early ROM) is an independent risk factor for cesarean delivery in nulliparous women who delivered after the spontaneous onset of labor [17]. It has been theorized that spontaneous early ROM is more likely in women with cephalopelvic disproportion, because the entire force of labor converges on the portion of the membranes that overlies the cervix in contracted pelvis that precludes the passage of the fetus [17], [18].

It is an important issue if there is a relationship between the occurrence of early ROM and the risk of cesarean delivery in induced labor, because prediction of the risk for cesarean delivery is not straightforward in clinical management of induced labor, which itself increases the risk of cesarean delivery[2], [5]–[10], and the timing of ROM is the kind of information that is readily available to physicians in the clinical setting. In addition, induced labor may be a better model for demonstration of a relationship between early ROM and risk of cesarean delivery than spontaneous onset of labor, because both the initiation of labor and ROM always occurs in the hospital during labor induction, resulting in a clear distinction between early ROM and late ROM.

To address this issue, we undertook this study to determine if spontaneous early ROM during induction of labor is associated with an increased risk for cesarean section in term nulliparas.

## Methods

### Study Design

In this retrospective cohort study, the rate of cesarean section and the timing of ROM during the course of labor were determined in term singleton nulliparas who were admitted for induction of labor between March 2002 and December 2007. Cases were classified into either an “early ROM” group or “late ROM” group according to the timing of ROM. Cases were extracted from the database of Seoul National University Hospital. Patients in whom the onset of labor or ROM occurred spontaneously before the induction of labor or those with an intrauterine fetal demise were excluded. The collection and the use of clinical data and the results of placental histologic examination for research purpose were approved by the institutional review board of Seoul National University Hospital.

### “Early ROM” and “Late ROM”

“Early ROM” and “late ROM” was defined as previously described [17]. In brief, “early ROM” was defined as spontaneous ROM before the onset of active labor (ROM before a cervical dilatation of 4 cm) during the course of labor, and “late ROM” was defined as ROM not occurring before the onset of active labor (i.e. ROM occurring at a cervical dilatation of 4 cm or more, either spontaneous or artificial). The cases with pre-labor ROM, defined as ROM in the absence of labor, after the induction of labor were excluded from analysis. Labor was defined as painful regular uterine contractions resulting in cervical change after the induction of labor. The partogram of the labor course, timing of ROM and the nature of ROM (spontaneous vs. artificial) are routinely documented in medical records in our institution.

### Clinical Management

Labor induction was performed with prostaglandins (dinoprostone or misoprotsol), intravenous oxytocin, or a combination of these agents. Oxytocin was administered intravenously as a dilute solution by infusion pump, and high-dose regimen is usually used; initial dose was started at 5.3 mU/min and it was increased every 15–30 minutes up to a maximum dose of 40 mU/min, with modification individualized to patient at the discretion of attending physician. Although the decision to perform an amniotomy was at the discretion of the attending physician, elective early amniotomy is not a routine practice in our institution. The diagnosis of failure to progress or fetal distress and the decision to perform cesarean section during the course of labor was made in accordance with the ACOG recommendations [19], [20].

### Acute Histologic Chorioamnionitis

Acute histologic chorioamnionitis was defined as the presence of acute inflammatory changes on examination of a membrane roll and chorionic plate of the placenta; funisitis was diagnosed as the presence of neutrophil infiltration into the umbilical vessel walls or Wharton’s jelly, according to the criteria previously described in detail [21].

### Statistical Analysis

Proportions were compared with Fisher’s exact test and comparisons of continuous variables between groups were performed with the Mann-Whitney U test. To assess which variables were significantly associated with cesarean section, multiple logistic regression analysis with backward elimination was conducted. The confounding variables in multiple logistic regression analysis were selected according to the analysis of univariate analysis as risk factors for cesarean section (p<0.2) [22]. A P-value<0.05 was considered significant.

## Results

### Characteristics of the Study Population

During the study period, there were a total of 5,481deliveries of singleton gestations. Among these, 4,603 cases were delivered at term, including 2,480 cases of nulliparas. Among these term singleton nulliparas, 565 cases underwent induction of labor before spontaneous onset of labor or ROM, without an intrauterine fetal demise. The timing of ROM was not available in the medical records of 10 cases. The remaining 555 cases were classified into either an “early ROM” group or “late ROM” group according to the timing of ROM, and additional 55 cases were excluded from analysis (39 cases with pre-labor ROM and 16 cases in whom the distinction between early ROM and late ROM was not possible (13 cases in whom emergent cesarean section was performed before a cervical dilatation of 4 cm with intact membranes, and 3 cases because artificial ROM was performed before a cervical dilatation of 4 cm)). Finally, five hundred cases were eligible for study and Table 1 lists the indications for labor induction in the study population.

**Table 1:** Indications for labor induction in the study population.

Indications for labor induction	Number of cases
Advanced gestational age	250
Hypertensive diseases in pregnancy	71
Oligohydramnios	55
Small for gestational age	37
Large for gestational age	9
Progressing fetal anomaly	9
Diabetes	17
Maternal medical disease	28
Non-reassuring fetal status	5
Others	19
Total	500

“Early ROM” occurred in 43% (215/500) and “late ROM” occurred in 57% (285/500) of the patients. Table 2 compares the demographic and intrapartum characteristics of the study population according to the timing of ROM. There were no significant differences in the clinical characteristics, including pre-pregnancy BMI, proportion of complicated pregnancies, cervical dilatation at the time admission, duration of labor, and use of prostaglandins or regional anesthesia between the two groups of cases. However, women with “early ROM” were of advanced maternal age and had a longer duration of ROM, and more frequent use of intravenous oxytocin than those with “late ROM” (p<0.05 for each).

**Table 2:** Demographics and intrapartum characteristics.

Characteristics	Early ROM (n = 215)	Late ROM (n = 285)	P value
Maternal age (years)†	31±4	30±4	0.03
Pre-pregnancy BMI (kg/m^2^)†	20.9±2.7 (available in 203 women)	21.1±3.1 (available in 273 women)	NS
Hypertensive disease in pregnancy	36 (17%)	33 (12%)	NS
Diabetes	11 (5%)	17 (6%)	NS
Fetal anomalies	19 (9%)	22 (8%)	NS
Cervical dilatation at admission	0.4±0.5	0.4±0.5	NS
Artificial ROM	0 (0%)	103 (36%)	<0.01
Duration of ROM (hours)†	10.9±11.1	2.0±5.3	<0.01
Total duration of labor (hours)†,‡	4.5±6.8	3.7±3.1	NS
Duration of 1^st^labor (hours)†,‡	3.2±6.7	2.6±2.7	NS
Duration of 2^nd^ labor (hours)†,‡	1.3±1.4	1.1±1.1	NS
Vaginal prostaglandins	210 (98%)	273 (96%)	NS
Intravenous oxytocin	188 (87%)	203 (71%)	<0.01
Regional analgesia	134 (62%)	155 (54%)	0.08

ROM: rupture of membranes, BMI: body mass index.

† Values are given as the mean ± standard deviation.

‡ Duration of 1^st^ stage of labor was defined as the duration of cervical dilatation from 4 cm to 10 cm; duration of 2nd stage was defined as duration between full cervical dilatation and fetal delivery; total duration of labor was defined as the sum of duration of 1^st^ and 2^nd^ stages of labor.

### Pregnancy Outcomes

Table 3 summarizes the pregnancy outcomes for the two groups. There were no significant differences in gestational age at delivery, and birthweight or proportion of macrosomia (birthweight>4 kg) between the two groups. The overall cesarean section rate was significantly higher in patients with “early ROM” than in those with “late ROM” (24% [51/215] vs. 10% [28/285]; p<0.01), and this difference remained significant after adjusting for maternal age, use of intravenous oxytocin or regional analgesia, gestational age at delivery, birthweight, macrosomia, and presence of acute histologic chorioamnionitis and funisitis (adjusted odds ratio (OR), 2.35; 95% Confidence interval (CI), 1.31–4.20; see Table 4, These confounding variables were selected according to the analysis of univariate analysis as risk factors for cesarean section (p<0.2)).

**Table 3:** Pregnancy outcomes.

	Early ROM(n = 215)	Late ROM (n = 285)	P value (unadjusted)	P value (adjusted) *
Gestational age at delivery(wks)†	40.1±1.4	40.3±1.2	NS	(−)
Birthweight(g)†	3285±461	3245±499	NS	(−)
Macrosomia	9 (4%)	18 (6%)	NS	NS
Overall cesarean section	51 (24%)	28 (10%)	<0.01	<0.01
C/S due to FTP	38 (18%)	22 (8%)	<0.01	0.02
C/S due to fetal distress	10 (5%)	6 (2%)	NS	NS
Operative vaginal delivery§	61/164 (37%)	76/257(30%)	NS	NS
Acute histologic chorioamnionitis	42/201 (21%)	46/258 (18%)	NS	NS
Funisitis	8/201 (4%)	14/258 (5%)	NS	NS
1-min Apgar score <7	17 (8%)	36 (13%)	NS	NS
5-min Apgar score <7	8 (4%)	9 (3%)	NS	NS

FTP: failure to progress.

† Values are given as the mean ± standard deviation.

* Adjusted for maternal age, presence of hypertensive disease in pregnancy, use of intravenous oxytocin or regional analgesia (logistic regression analysis).

§ Analyzed only in cases who delivered vaginally.

Among cases with cesarean section, 76% (60/79) of cesarean sections were performed due to failure to progress. The rate of cesarean sections due to failure to progress was also significantly higher in patients with “early ROM” than those with “late ROM” (18% [38/215] vs. 8% [22/285]; p<0.01) and this difference also remained significant after adjusting for maternal age, use of intravenous oxytocin or regional analgesia, gestational age at delivery, birthweight, macrosomia, and presence of acute histologic chorioamnionitis and funisitis [adjusted odds ratio (OR), 2.06; 95% Confidence interval (CI), 1.06–3.99]. However, the rate of cesarean section due to fetal distress and the rate of operative vaginal deliveries were not different between the two groups (p>0.1 for each, Table 3).

### Cases whose Membranes were Spontaneously Ruptured

Among the study population, spontaneous ROM occurred in 397 cases. In cases of “late ROM”, spontaneous ROM occurred in 182 cases (64%) and artificial ROM was performed in 103 cases (36%). The cesarean section rate was not different between cases with spontaneous ROM and those with artificial ROM (16% vs. 14%, p = NS). Even when confining the analysis to these cases with spontaneous ROM (i.e. after excluding cases with artificial ROM), the rate of overall cesarean section and the rate of cesarean section due to failure to progress was higher in cases with early ROM than those with late ROM (overall cesarean section rate: 24% [51/215] vs. 8% [14/182], p<0.01; cesarean section rate due to failure to progress: 18% [38/215] vs. 7% [12/182], p<0.01). This difference remained significant after adjusting for confounding variables (p<0.01 for overall cesarean section and p<0.05 for cesarean section due to failure to progress).

## Discussion

The principal findings of this study were: 1) “early ROM” occurred in 43% in term singleton nulliparas after induction of labor; 2) patients with “early ROM” had a higher rate of overall cesarean section and cesarean section due to failure of progress than did those with “late ROM” (p<0.01 for each) and this difference remained significant after adjustment for confounding variables.

Our study demonstrated that early ROM can be considered as a risk factor for cesarean delivery in induced labor, in addition to other risk factors including unfavorable cervix, advanced maternal age, epidural analgesia, intravenous oxytocin, macrosomia, and PROM[23]–[26]. In the literature, we were not able to find any reports which examined the relationship between the timing of ROM during induction and the risk of cesarean delivery in induced labor. This relationship is important because the timing of ROM is information that is readily available to clinicians and predicting the risk for cesarean delivery is clinically important issue. In addition, early ROM could be added as a new risk factor into scoring systems[12]–[16], which were developed to predict the likelihood of requiring cesarean delivery.

Among eligible populations, cases with pre-labor ROM during the course of induction were excluded (39 of 595 cases). The overall cesarean section rate in cases with pre-labor ROM was 17.9% (7/39), which was less than that in cases with early ROM and higher than that in cases with late ROM (p<0.001, χ^2^ test for trend). Thus, cases with early ROM had the highest cesarean section rate among the three groups (pre-labor ROM, early ROM, and late ROM).

Several explanations for this relationship between early ROM and the risk of cesarean delivery in induced labor may be offered. First, early ROM itself may be reflective of dystocia with cephalopelvic disproportion, as suggested in our previous report [17]. Vrouenraets et al [23] demonstrated that induction of labor in nulliparas is associated with an increased risk of cesarean delivery, predominantly related to an unfavorable Bishop score on admission, and no significant differences in the rate of cesarean delivery existed among groups with induced labor and spontaneous labor after adjustment for the Bishop score. An unfavorable cervix as a risk factor for cesarean delivery in induced labor was also reported by the study of Johnson et al. [24]. It is possible that early ROM may reflect the possible mechanism (dystocia) of this relationship between unfavorable cervix and increased risk of cesarean delivery. In induced labor, it is possible that a relatively unripened cervix at the beginning of induction may contribute to “relative cephalopelvic disproportion” during subsequent labor progression, becoming an obstacle to the descent of fetal head. Unfavorable cervix in induced labor may result in relatively ineffective cervical dilatation in spite of medically-induced uterine contractions, then the fetal head may arrest in the pelvic inlet during labor. And the uterine contractile forces may focus on the presenting fetal membranes, resulting in early ROM and resulting in increased risk of cesarean section. This relationship between unfavorable cervix and early ROM can be also assumed from the fact that early ROM is more common in induced labor than in spontaneous onset of labor. In the current study, early ROM occurred in 43% of cases with induced labor. This proportion is higher than that in term nulliparas with spontaneous onset of labor, which was reported in our previous report [17] (24% [109/447]), although direct comparison should be interpreted with caution considering possible differences between these two populations (women with spontaneous onset of labor vs. women with induced labor). Why is early ROM in induced labor more common than, or at least comparable to, that in spontaneous onset of labor? Reminding that early ROM itself may be suggestive of dystocia with cephalopelvic disproportion [17], it is possible that a relatively less ripened cervix in induced labor than in spontaneous onset of labor may contribute to “relative cephalopelvic disproportion”, resulting in early ROM. However, further studies are needed to address this mechanism, because we were not able to demonstrate the relationship between bishop score and early ROM because of the absence of data on bishop score in the study population.

Second, the absence of hydrostatic pressure of membranes after ROM may result in slow progress of labor and a corresponding increased risk of cesarean delivery. During labor with intact membranes, uterine contractile forces exert pressure on the fetal membranes, resulting in centrifugal force on the cervix and cervical dilatation [27]_ENREF_17, whereas in cases with early ROM, this mechanism may be interrupted because of absent fetal membranes. However, the duration of labor was not different between cases with early ROM and those with late ROM in the current study, refuting this explanation.

Third, intra-amniotic infection or inflammation may result from a longer duration of ROM in cases with early ROM and are responsible for an increased risk of cesarean delivery because of inadequate uterine contractions due to uterine inflammation. It has been suggested that high virulence bacterial infections or chorioamnionitis are associated with dystocia[28]–[30]. However, the relationship between early ROM and the risk of cesarean delivery remained significant even after adjustment for acute histologic chorioamnionitis and funisitis in the current study (Table 4). In addition, the rate of histologic chorioamnionitis and funisitis in the current study was 19% (88/459) and 5% (22/459), respectively. This is comparable to the frequency of histologic chorioamnionitis and funisitis in previous reports at term pregnancy [31], [32].

**Table 4:** Relationship of various independent variables with the risk of cesarean section analyzed by multiple logistic regression analysis with backward elimination.

Variables	Odds ratio	95% Confidence interval	P value
Early ROM	2.35	1.31–4.20	<0.01
Maternal age (years)	1.14	1.05–1.23	<0.01
Oxytocin augmentation	3.23	1.25–8.32	0.02
Birthweight	1.00	1.00–1.002	<0.01
Acute histologic chorioamnionitis	2.63	1.34–5.14	<0.01
Funisitis	4.25	1.42–12.74	0.01

ROM: rupture of membranes, BMI: body mass index, NS: not significant.

It was reported that several factors were associated with the increased risk of cesarean section during the induction of labor. First, previous studies have demonstrated that lower bishop score was associated with increased risk of cesarean delivery [24], [33], [34]. In the current study, we were not able to analyze the effect of bishop score on the rate of cesarean delivery, because the bishop score was not always available in the study population. Instead, the cervical dilatation before labor induction was analyzed, and the relationship between the occurrence of early ROM and the risk of cesarean delivery remained significant even after adjustment for cervical dilatation at admission. Second, several studies have issued the indication of labor induction as a risk factor for cesarean delivery[35]–[37]. In the current study, induction of labor was performed according to various indications (Table 1). However, early ROM was a significant risk factor for cesarean delivery, even after adjustment for the indication of labor induction (data not shown). Third, the higher rate of the use of intravenous oxytocin in patients in early ROM group than in those in late ROM group in this study might be associated with the increased rate of cesarean section. However, the occurrence of early ROM was significantly associated with increased odds of cesarean section even after adjustment of other confounding variables including the use of intravenous oxytocin (Table 4).

In the spontaneous onset of labor of nulliparas, early ROM has been demonstrated as a risk factor for cesarean delivery [17]. To demonstrate a relationship between early ROM and risk of cesarean delivery, induced labor may be a good model because the initiation of labor and ROM occurs in the hospital during labor induction, resulting in a clear distinction between early ROM and late ROM. Indeed, this distinction between the two groups was not available in only 10 cases among the study population in the current study (1.96% [10/510]), whereas this distinction was not available in 6.49% (31/478) of cases with spontaneous onset of labor in a previous report [17].

In conclusion, early ROM during labor induction is associated with an increased risk of cesarean delivery. Further studies on the possible mechanism of this association and on the development of new scoring system using early ROM for prediction of cesarean section in induced labor will be needed to enhance our understanding on the nature of early ROM in induced labor.
